# Expressed sequence tag analysis of adult human optic nerve for NEIBank: Identification of cell type and tissue markers

**DOI:** 10.1186/1471-2202-10-121

**Published:** 2009-09-24

**Authors:** Steven L Bernstein, Yan Guo, Katherine Peterson, Graeme Wistow

**Affiliations:** 1 Departments of Ophthalmology and Neurobiology & Genetics, University of Maryland School of Medicine, Baltimore, Maryland USA; 2 Section on Molecular Structure and Functional Genomics, National Eye Institute, National Institutes of Health, Bethesda, Maryland USA

## Abstract

**Background:**

The optic nerve is a pure white matter central nervous system (CNS) tract with an isolated blood supply, and is widely used in physiological studies of white matter response to various insults. We examined the gene expression profile of human optic nerve (ON) and, through the NEIBANK online resource, to provide a resource of sequenced verified cDNA clones. An un-normalized cDNA library was constructed from pooled human ON tissues and was used in expressed sequence tag (EST) analysis. Location of an abundant oligodendrocyte marker was examined by immunofluorescence. Quantitative real time polymerase chain reaction (qRT-PCR) and Western analysis were used to compare levels of expression for key calcium channel protein genes and protein product in primate and rodent ON.

**Results:**

Our analyses revealed a profile similar in many respects to other white matter related tissues, but significantly different from previously available ON cDNA libraries. The previous libraries were found to include specific markers for other eye tissues, suggesting contamination. Immune/inflammatory markers were abundant in the new ON library. The oligodendrocyte marker QKI was abundant at the EST level. Immunofluorescence revealed that this protein is a useful oligodendrocyte cell-type marker in rodent and primate ONs. L-type calcium channel EST abundance was found to be particularly low. A qRT-PCR-based comparative mammalian species analysis reveals that L-type calcium channel expression levels are significantly lower in primate than in rodent ON, which may help account for the class-specific difference in responsiveness to calcium channel blocking agents. Several known eye disease genes are abundantly expressed in ON. Many genes associated with normal axonal function, mRNAs associated with axonal transport, inflammation and neuroprotection are observed.

**Conclusion:**

We conclude that the new cDNA library is a faithful representation of human ON and EST data provide an initial overview of gene expression patterns in this tissue. The data provide clues for tissue-specific and species-specific properties of human ON that will help in design of therapeutic models.

## Background

The optic nerve (ON) is an isolated CNS tract, supplied by a separate vasculature, that connects the eye to the rest of the central nervous system (CNS). The ON consists of the myelinated axons of retinal ganglion cells (RGC), their supporting glia, oligodendrocytes and vascular elements, all enclosed by a fibrous sheath. The ON is one of the few areas that a pure CNS white matter tract is readily available for analysis, providing a window into in-vivo CNS axonal function. In humans, the 8 cm long ON is clinically subject to a number of diseases, notably the glaucomas, optic neuritis and anterior ischemic optic neuropathy (AION)[1]. Relatively little is known about gene expression patterns in human ON and their implications for ON-specific disease, or about species-specific differences in gene expression that may contribute to the dichotomies in pharmacological responsiveness known to occur between humans and rodent models of CNS disease [2,3]. In addition, the ON provides a near-ideal tool for identifying axonally transported mRNAs; a newly described neuronal function [4,5].

Expressed sequence tag (EST) analysis of cDNA libraries can provide an informative overview of major transcripts in specific tissues. The NEIBank project has created and analyzed several cDNA libraries from specific eye tissues [6-9]. While many cDNA libraries are normalized (a subtraction hybridization approach to reduce the representation of abundant clones) or amplified (an expansion in which different clones proliferate at different rates), most NEIBank libraries are unnormalized and unamplified so that random sequencing for EST analysis reflects more closely the natural abundance of common gene transcripts in each tissue. This information can shed light on the molecular bases for the structural and functional differences among tissues, and for important differences in tissue responsiveness to pharmacological agents and sensitivity to various pathological processes.

EST data described as originating from human optic nerve is available in the Unigene database (Unigene Libraries10279 and 10284). However inspection of the data suggested that these libraries may be mis-identified and may not actually represent optic nerve, or may be grossly contaminated with other tissues. Here the construction and analysis of a new unnormalized human ON library is described. The new library shows strong similarities in gene expression to other neural tissues while previously available Unigene data contains markers for anterior segment and retina. The new analysis has revealed the expression of several genes with implications for ON function and with potential value as markers for specific cell types in the ON. The new ON library thus provides both a reasonable indicator of the pattern of gene expression in human ON. Results of this analysis also provide some insights into the variability of responsiveness to neuroprotective treatments exhibited by rodents and primates ON.

## Results and Discussion

### cDNA Library and Sequencing

For the human ON cDNA library (*nbj*), there were 2.2 × 10^6 ^primary transcripts, with an average insert size of 1.3 kbp. 2.2% of clones contained no insert and 6% contained mitochondrial genome sequence. A total of 4651 quality 5' reads from the library yielded 4269 clones after removal of contaminants and very short sequences and masking of repetitive sequences. Analysis of these clones using GRIST [10] resulted in identification of 2789 groups of clones, each potentially representing individual ON expressed genes. 375 of these groups contained two or more clones. These results enable us to generate a 'first pass' analysis of about the relative expression of the more common genes, and allow us to compare characteristics of different CNS white matter libraries.

About 75% of the groups of clones corresponded to identified genes with corresponding RefSeq or Unigene entries in GenBank. The remainder consists of singleton clones, many of which represent longer 3' UTRs of known genes, sequences not include in RefSeq, sequences with high Phred quality scores that nevertheless have insufficiently good sequence to give a reliable match, and clones from intron and intergenic regions. The latter class may or may not have biological significance, although it is now clear that much more of the genome is transcribed than was previously thought [11,12]. The position of such clones can be examined using tools in NEIBank and EyeBrowse [13]. Table 1 shows the most abundant expressed genes the human ON library (top 36), those represented by 5 or more clones in the EST analysis.

**Table 1:** Most abundant ESTs from un-normalized human ON library.

**GenBank Description**	**Gene Id**	**Genome Position**	**#**
glial fibrillary acidic protein (GFAP)	2670	chr17:40338955	47
proteolipid protein 1(PLP1)	5354	chrX:102928226	41
clusterin (CLU)	1191	chr8:27511430	40
myelin basic protein (MBP)	4155	chr18:72820816	33
eukaryotic translation elongation factor 1 alpha 1 (EEF1A1)	1915	chr6:74284050	29
SPARC-like 1 (hevin) (SPARCL1)	8404	chr4:88619666	12
secreted phosphoprotein 1 (osteopontin) (SPP1)	6696	chr4:89120428	11
ferritin, heavy polypeptide 1 (FTH1)	2495	chr11:61488752	10
glutamine synthetase (GLUL)	2752	chr1:180618678	10
prostaglandin D2 synthase (PTGDS)	5730	chr9:138991792	9
**vimentin (VIM)**	7431	chr10:17312655	9
**beta actin (ACTB)**	60	chr7:5533613	8
secreted protein, acidic, cysteine-rich (osteonectin) (SPARC)	6678	chr5:151023929	8
neurotrophic tyrosine kinase, receptor, 2 (NTRK2)	4915	chr9:86619050	7
quaking homolog (QKI)	9444	chr6:163908138	7
ribosomal protein L3 (RPL3)	6122	chr22:38040726	7
ribosomal protein L4 (RPL4)	6124	chr15:64580417	7
calmodulin 2 (CALM2)	805	chr2:47240949	6
carboxypeptidase E (CPE)	1363	chr4:166638437	6
prosaposin (PSAP)	5660	chr10:73246834	6
serpin peptidase inhibitor A3 (alpha-1 antitrypsin) (SERPINA3)	12	chr14:94159798	6
annexin A1 (ANXA1)	301	chr9:74963260	5
apolipoprotein D (APOD)	347	chr3:196776979	5
CD74 (MHC class II invariantchain) (CD74)	972	chr5:149761850	5
dystonin (DST)	667	chr6:56431813	5
heat shock 22 kDa protein (HSPB8)	26353	chr12:118101133	5
integral membrane protein 2B (ITM2B)	9445	chr13:47730267	5
myosin, light chain 6 (MYL6)	4637	chr12:54838422	5
reticulon 4 (RTN4)	57142	chr2:55053469	5
ribosomal protein L10 (RPL10)	6134	chrX:153282028	5
septin 7 (SEPT7)	989	chr7:35888660	5
**ubiquitin C (UBC)**	7316	chr12:123963520	5

First column: Gene name. Column two indicates the gene ID number from Genbank. Genome position on each chromosome is shown in the third column and the number of ESTs for each gene is shown in the fourth column.

### Comparisons with related datasets

Prior to this new EST analysis, two publically available datasets for cDNA libraries also described as human ON were available through Unigene (Unigene Lib.10279 (unnormalized), Lib.10284 (normalized from the same source). Supplemental Table S1 [see Additional file 1] shows the most abundant groups of cDNAs from the unnormalized data. It is immediately apparent that markers for anterior segment, such as keratin 12 and opticin, and markers for retina, such as rhodopsin and α-transducin, are abundantly represented in this dataset. Other anterior segment and retina markers, such as ALDH3A1, aquaporin 5, cadherin 23 and retinol binding protein 3 (IRBP) are also present at lower levels. This suggests that the tissue origin of these libraries is probably not isolated optic nerve and the libraries are at least contaminated with retina and anterior segment. Indeed, this observation was a major reason for the creation of the present ON library for NEIBank. Data from the combined Unigene libraries have been processed at NEIBank as a resource of eye expressed clones, but because of the uncertainty about tissue origin this data is listed as "For the Record" rather than as ON.

In contrast to the Unigene library data, EST analysis of the new NEIBank ON library (*nbj*) shows abundant markers characteristic for white matter neural tissue while retina and anterior segment markers (such as rhodopsin and opticin) are absent from *nbj*. Four genes expressed by oligodendrocytes (myelin-associated oligodendrocyte basic protein, prosaposin, QKI, and reticulon-4 (NOGO)) that would be expected to be highly expressed in any library generated from ON are present in *nbj*, but are absent or low abundance in the Unigene data. Indeed, of the 134 genes represented by 3 or more ESTs in the new ON library, 107 (80%) are absent from the unnormalized Unigene library.

**Figure 1:**
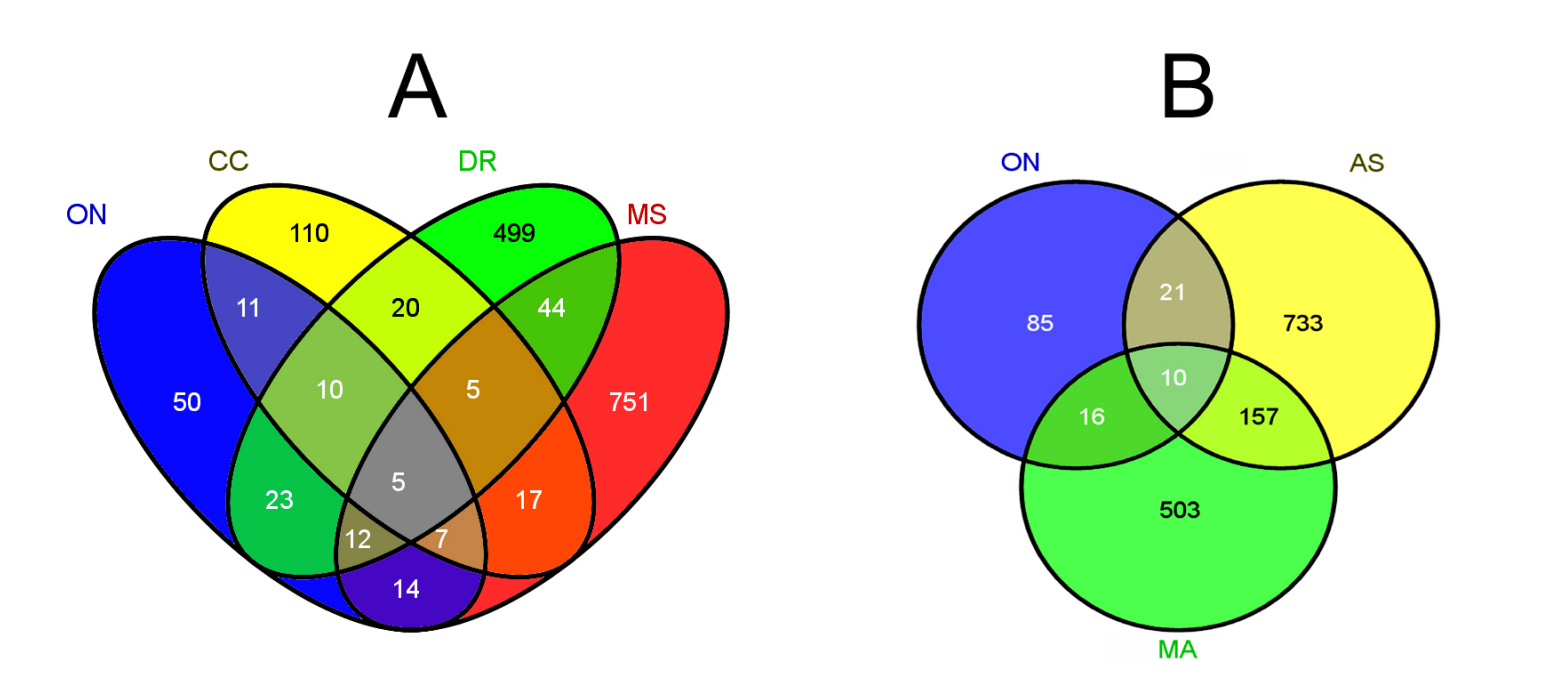
**Comparison of abundantly expressed (more than 3 ESTs) genes in ON, compared with abundantly expressed genes in other libraries representing white matter, astrocytes and macrophages**. A) CC: human corpus callosum (dbEST:16383). MS: Multiple sclerosis lesions (dbEST: 390). DR: Human dorsal root ganglion (dbEST:5655). B) MA: Human purified macrophages (dbEST:16419). AS: human purified astrocytes (dbEST:18304).

ON is a CNS-white matter tract that also contains large numbers of astrocytes and microglia (intrinsic macrophages). For tissue comparison, we evaluated EST data for white matter neural tissues, including corpus callosum (CC; the axonal fiber tract connecting the two cerebral hemispheres, dbEST:16383), a library generated from white matter-affected multiple sclerosis lesions (dbEST: 390) and an un-normalized dorsal root ganglion (dbEST:5655). Additionally, we included EST library data from astrocytes (dbEST:18304), and macrophages (dbEST:16419), extracted from UniGene. While the multiple sclerosis library gene abundance is normalized, the other datasets are un-normalized. Since representation of low abundance genes in EST data is stochastic, the comparisons were limited to "abundant' genes, simply defined as those represented by 3 or more clones in a library. Figure 1 shows the comparison of the abundant clones in the ON data with those in the neural and with the astrocyte/macrophage libraries (based on Unigene assemblies). About 60% of the genes abundant in ON are also abundant in the other white matter libraries while about 40% are also abundant in astrocytes or macrophages. 42 of the abundant genes in ON are abundant in neither of the other groups. Many of these have undefined or general functions. Those listed in Table 2 have potentially interesting connections to functional roles in ON. Two of the genes code for dystonin and dynein, proteins which are known to interact [14]. Another interesting gene in this category is EFEMP1/Fibulin3, an ECM protein of unknown function that is mutated in the AMD-like disease Malattia leventinese/Doyne honeycomb retinal dystrophy (ML/DHRD). Other genes in this list have known roles in oligodendrocytes, cytoskeleton or cellular motors. Some map to areas associated with inherited glaucoma or retinal disease (for an overview of these regions, use the Candidate Disease Region page at the NEIBank web site).

**Table 2:** Selected genes abundant in ON but not in other white matter/astrocyte/macrophage libraries.

**UniGene**	**UniGene Description**	**#**	**Notes**
121333	Myelin-associated oligodendrocyte basic protein	5*	Oligodendrocyte
631992	Dystonin	5	cytoskeleton anchor/RP25 and BCMAD locus
632717	Myosin, light chain 6	5	cellular motor protein
76224	EFEMP1	4^	Doyne honeycomb retinal dystrophy/GLC1H locus
422181	S100 calcium binding protein B	4	oligodendrocytes maturation
58414	Filamin C, gamma	3	actin cytoskeleton
117060	Extracellular matrix protein 2	3	GLC1J locus
151220	Palladin	3	cytoskeletal remodeling
369068	Dynein, cytoplasmic 1, light intermediate chain 2	3	microtubule motor/interacts with dystonin
501140	KIAA1598/Shootin	3	neuronal polarization
528087	Inositol 1,4,5-trisphosphate 3-kinase B	3	Ca2+-leak
533683	Fibroblast growth factor receptor 2	3	optic atrophy

The first column shows the unigene number, the second column the gene descriptor. Third column indicates the number of specific ESTs in the first 2000 sequenced clones. The fourth column (notes) give specific information of interest about these genes * A possible 6^th ^clone in 3'UTR is also present. ^ A 5^th ^clone was dropped because it is chimeric.

### ON expressed with genes with functional implications

#### Axon-associated

RGC axons are major components of ON. One of the more abundantly expressed axonal genes in ON is dystonin or BPAG1, represented by five ESTs. Dystonin is a plakin family member regulated by IFNγ and is associated with retrograde axonal transport in sensory neurons [15]. As mentioned above, this gene is not abundantly expressed in a sample of other white matter-related libraries. Another axonally expressed gene that is associated with axon outgrowth and axon-dendrite specification, dihydropyrimidinase-like 2 (DPYSL2), also known as collapsin response mediator protein 2 [16] is represented by 4 ESTs, while SEMA3B, a gene associated with axon guidance has 3 ESTs in the collection. Other genes with roles in axonal growth and function, such as dynein subunits and ROBO3, are also represented at lower levels.

#### Putative axonally-transported mRNAs

Axon transport of mRNAs has been shown in a number of animal models. A number of proteins associated with cytoskeleton, injury-response, and neurodegeneration are specially transported to and translated in axons. We compared reported axonally-transported mRNAs with the human equivalent ESTs identified in the new human ON cDNA library (*nbj*). Table 3 shows the concordance in human ON with known axonally transported mRNAs. The human homologs of 18/26 genes (69%) demonstrated to be axonally transported in rat dorsal root ganglion were identified in the ON library. ESTs for Vimentin and *beta*-actin were among the highest abundance in the un-normalized library, with 8 other ESTs present in multiple copies. While a number of ESTs identified in this group are also generally expressed in many cell types (for example, HSP90, GRP78 and Glyceraldehyde 3-phosphatase), these data suggest that mRNA axonal transport is likely to be a common aspect of human CNS axonal function as well.

**Table 3:** Genes expressed in ON that are associated with axonal synthesis.

**Description**	**Gene ID**	**#**
vimentin (VIM)	7431	9
actin, beta (ACTB)	60	8
actin, gamma 1 (ACTG1)	71	4
peptidylprolyl isomerase A (cyclophilin A) (PPIA)	5478	4
glyceraldehyde-3-phosphate dehydrogenase (GAPDH)	2597	4
enolase 1, (alpha) (ENO1)	2023	4
heat shock protein 90 kDa alpha B1 (HSP90AB1)	3326	3
peroxiredoxin 1 (PRDX1)	5052	3
Parkinson disease (autosomal recessive, early onset) 7 (PARK7)	11315	2
heat shock 60 kDa protein 1 (chaperonin) (HSPD1)	3329	2
tropomyosin 3 (TPM3)	7170	1
aldolase C, fructose-bisphosphate (ALDOC)	230	1
superoxide dismutase 1 (SOD1)	6647	1
cofilin 1 (non-muscle) (CFL1)	1072	1
synuclein, gamma (SNCG)	6623	1
peroxiredoxin 6 (PRDX6)	9588	1
calreticulin (CALR)	811	1
heat shock 70 kDa protein 5 (HSPA5)	3309	1

Column 1: gene name. Column 2: Gene ID. Column 3: relative abundance of each identified EST, from the first 2000 sequenced ESTs.

#### Signaling pathways

ESTs for several genes associated with intracellular signaling are also represented abundantly in nbj. These include mitogen-activate protein kinase-kinase kinase 13, neurotrophic tyrosine kinase receptor type 2, and calmodulin 2. Other signaling components such as FGFR2, STAT1, kinectin 1 and MAPK1 are also represented by multiple ESTs.

#### Oligodendrocyte markers

One of the most abundant ON expressed genes in the *nbj *analysis is QKI (quaking homolog). There are 7 ESTs for QKI, but it is absent from both the unnormalized and normalized Unigene datasets). QKI is an oligodendrocyte-specific gene expressed in both cytoplasm and nucleus. QKI isoforms are dramatically reduced or absent in twitcher mice, and are required for normal myelination [17]. We tested QKI as a nuclear marker for ON-oligodendrocyte identification. A rabbit polyclonal anti-QKI antibody was reacted against rat, monkey and human ON, and compared with immunoreactivity with adenoma polyposis coli (APC-1), which has been identified as selectively reacting with oligodendrocyte and astrocyte nuclei [18], as well as against rat and human retina.

**Figure 2:**
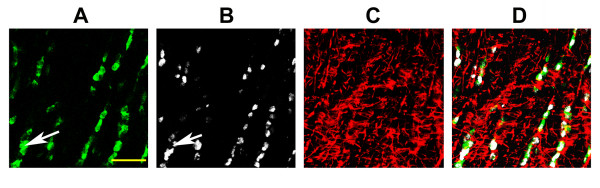
**ON immunolocalization of oligonucleotide and astrocyte selective proteins**. **A: APC-1, B: QKI, C: GFAP, D: Merged confocal image**. APC-1 and QKI show considerable overlap of immunoreactivity (compare A and B and merged image, D). Few cells stain either with APC-1 alone (arrow) or QKI. APC-1/QKI positive astrocytes can be distinguished using colocalization with GFAP (compare panels C and merged image, D). Scale bar: 50 microns.

In all species the anti-QKI antibody generated a strong nuclear signal in the columnar nuclei that typically are associated with oligodendrocytes (figure 2). Similar patterns were seen for anti-QKI antibody (figure 2B) and APC-1 antibody (figure 2A), another marker for oligodendrocytes [19] while anti-GFAP (figure 2C) stained both oligodendrocytes and astrocytes. Thus, in conjunction with GFAP, QKI can be used to selectively identify oligodendrocytes.

#### Ion-channels

**Figure 3:**
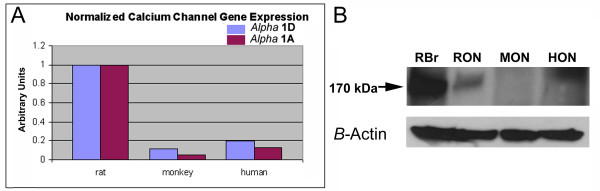
**RQ-PCR analysis of L-type calcium channel transcripts in ON, and corresponding ON-protein expression**. **A. RQ-PCR analysis**. Gene-specific primers for the α1A and α1D subunits of the L-type calcium subunits were generated from Genbank. RQ-PCR results were internally normalized using primers for cyclophilin. There is 7-10 fold less α1A and 5-7 fold less α1D mRNA in human and rhesus macaque ON than in rat ON. **B. Western analysis**. ON homogenates from rat brain (RBr), rat ON (RON), monkey ON (MON) and human ON (HON) were subjected to PAGE, transferred to PVDF membrane, and probed with a mouse monoclonal antibody to the α1D subunit of the L-type calcium channel. The specific protein (170kD; arrow) is detectable as a strong band present in the rat brain and -ON homogenates. Signal strength for the α1D subunit is considerably less in homogenates from monkey and human ON. The lower inset band is relative β-actin signal from each lane.

Table 4 shows voltage gated and ligand-gated ion channel-related transcripts identified by GO terms in the *nbj *human ON analysis. A single clone for one of the subunits of an L-type sodium-calcium ion channel was observed. Such channels play important roles in maintaining rodent CNS calcium homeostasis [20] and in rodent optic nerve ischemia [21] and have been postulated to play roles in human CNS diseases, including stroke and spinal cord trauma [22]. However, while numerous studies have documented efficacy in rodent model systems, beneficial effects of L-type calcium ion channel blockers have proven less effective in human trials [3]. We evaluated the relative expression of L-type calcium channels in rodent and primate ON. Real time quantitative PCR (PCR) analysis was used to measure mRNA levels for two subunits (the *alpha-*1A and *alpha*-1D subunits) of L-type calcium ion channels in human, rhesus monkey and rat ON. In order to confirm that mRNA abundance differences translate into real protein concentration difference, we performed western analysis using an antibody to the *alpha*-1D subunit of the L-channel calcium channel. These results are shown in figure 3.

**Table 4:** Genes for voltage-gated and ligand-gated ion channels in the ON dataset.

**Description**	**Gene ID**	**#**
chloride intracellular channel 4 (CLIC4)	25932	4
glutamate receptor, ionotropic, AMPA 1 (GRIA1)	2890	2
voltage-dependent anion channel 3 (VDAC3)	7419	2
calcium-activated potassium channel beta 4 subunit (KCNMB4)	27345	1
chloride channel 3 (CLCN3)	1182	1
Chloride channel 4 (CLCN4)	1183	1
chloride channel 5 (CLCN5)	1184	1
chloride channel, nucleotide-sensitive, 1A (CLNS1A)	1207	1
chloride intracellular channel 5 (CLIC5)	53405	1
polycystic kidney disease 2-like 2 (PKD2L2)	27039	1
potassium channel tetramerisation domain containing 10 (KCTD10)	83892	1
potassium channel tetramerisation domain containing 12 (KCTD12)	115207	1
potassium channel tetramerisation domain containing 18 (KCTD18)	130535	1
potassium channel, subfamily K, member 12 (KCNK12)	56660	1
potassium channel, T2 (KCNT2)	343450	1
potassium large conductance calcium-activated channel, M, alpha1 (KCNMA1)	3778	1
purinergic receptor P2X, ligand-gated ion channel, 7 (P2RX7)	5027	1
voltage gated channel like 1 (VGCNL1)	259232	1
voltage-dependent anion channel 2 (VDAC2)	7417	1
voltage-gated sodium channel beta-1 subunit (SCN1B)	6324	1
Calcium channel, voltage-dependent, L type, alpha 1D subunit	476358	1

These are identified by GO terms: ATP-gated cation channel activity; Calcium-activated potassium channel activity; Extracellular-glutamate-gated ion channel activity; Ion channel activity; Potassium channel activity; Voltage-gated chloride channel activity; Voltage-gated ion channel activity; Voltage-gated ion-selective channel activity; Voltage-gated potassium channel activity; Voltage-gated potassium channel complex.

Results shown in figure 3 reveal that rat ON has a 5-7 fold higher abundance of tested L-type calcium ion channel transcripts than does old-world primate ON (figure 3A; compare channel gene expression of both isoforms in rat with that expressed in monkey and human). A qualitatively similar difference was also apparent at the protein level (figure 3B; western analysis). These results suggest that the difference in response to L-type calcium ion channel blockers in rodent and primate may be related to species-specific differences in gene expression in ON.

#### Immune/inflammatory markers

A significant component of ON consists of immune-related cells, particularly microglia. Three of the most abundant ESTs in *nbj*: CD74 (major histocompatibility complex, class II invariant chain), osteopontin, and prostaglandin D2 synthetase are associated with microglial-macrophage/inflammatory functions. Other abundant ESTs with functional connection to inflammatory processes are also present. SERPINA3 (alpha1-antichymotrypsin), represented by 6 ESTs, is an acute phase protein whose expression increases in acute and chronic inflammation and which may be involved in stroke and other neurological disease[23]. Annexin A1, represented by 5 ESTs is thought to have neuroprotective or anti-neuroinflammatory functions in brain [24]. An important caveat is that peri-mortem inflammatory conditions may influence the number of inflammation-associated ESTs in a human donor library.

#### Eye disease genes expressed in ON

Over 400 genes in which mutations or sequence variants directly affect vision are known (NEIBank ref). Many of these have clinical effects on ON. Table 5 shows the known eye disease genes for which ESTs are present in the *nbj *dataset. Several of these are known to be associated with ON disease. One abundantly expressed gene in *nbj *is EFEMP1/Fibulin3 (four ESTs) which is the locus for Malattia leventinese/Doyne honeycomb retinal dystrophy (ML/DHRD), an inherited disease with similarities to age-related macular degeneration [25]. This raises the question of whether there might be direct ON involvement in ML/DHRD. Interestingly, EFEMP1/Fibulin3 also happens to be the most abundant ON EST from the candidate gene region for a glaucoma, GLC1H (OMIM:611276).

**Table 5:** Eye Disease Genes with ESTs in ON.

**Gene**	**#**	**Disease**	**Omim**
PLP1	41	Pelizaeus-Merzbacher Disease	312080
CRYAB	5	Cataract, Posterior Polar, 2	123590
ITM2B	5	Dementia, Familial Danish	117300
EFEMP1	4	Doyne Honeycomb Retinal Dystrophy	126600
GJA1	4	Oculodentodigital Dysplasia	164200
FGFR2	3	Crouzon Syndrome	123500
DCN	2	Corneal Dystrophy, Congenital Stromal	610048
GSN	2	Corneal Dystrophy, Lattice Type1	122200
APC	1	Adenomatous Polyposis Of The Colon	175100
COL4A5	1	Alport Syndrome	301050
SOX2	1	Anophthalmos, True Or Primary	206900
BBS4	1	Bardet-Biedl Syndrome 4	209900
TTC8	1	Bardet-Biedl Syndrome 8	209900
CLN5	1	Ceroid Lipofuscinosis, Neuronal 5	256731
ARSE	1	Chondrodysplasia Punctata 1	302950
EBP	1	Chondrodysplasia Punctata 2, X-Linked Dominant	302960
VPS13B	1	Cohen Syndrome	216550
LRP2	1	Donnai-Barrow Syndrome	222448
ETHE1	1	Encephalopathy, Ethylmalonic	602473
GLA	1	Fabry Disease	301500
TIMP3	1	Fundus Dystrophy, Pseudoinflammatory, Of Sorsby	136900
MYOC	1	Glaucoma 1, Open Angle, A	137750
CYP1B1	1	Glaucoma 3, Primary Infantile, A	231300
FTL	1	Hyperferritinemia-Cataract Syndrome	600886
NDUFS7	1	Leigh Syndrome	256000
ASAH1	1	Macular Cherry-Red Spots	228000
APOE	1	Macular Degeneration, Age-Related, 1	603075
C2	1	Macular Degeneration, Age-Related, 1	603075
C3	1	Macular Degeneration, Age-Related, 9	611378
SNX3	1	Microcephaly, Microphthalmia, Ectrodactylyand Prognathism	601349
PAFAH1B1	1	Miller-Dieker Lissencephaly Syndrome	247200
GLB1	1	Mucopolysaccharidosis Type Ivb	253010
TRIM37	1	Mulibrey Nanism	253250
POMGNT1	1	Muscle-Eye-Brain Disease	253280
ACOX1	1	PEROXISOMAL ACYL-Coa OXIDASE DEFICIENCY	264470
PRPF8	1	Retinitis Pigmentosa 13	600059
PRPF3	1	Retinitis Pigmentosa 18	601414
ROM1	1	Retinitis Pigmentosa 7	608133
GNPAT	1	Rhizomelic Chondrodysplasia Punctata, Type 2	222765
NPHP3	1	Senior-Loken Syndrome 3	606995
NPHP4	1	Senior-Loken Syndrome 4	606996
GM2A	1	Tay-Sachs Disease, ab variant	272750
USH1C	1	Usher Syndrome, Type Ic	276904
DFNB31	1	Usher Syndrome, Type 2d	607084
POMT2	1	Walker-Warburg Syndrome	236670
PEX1	1	Zellweger Syndrome	214100

The gene name is shown in the first column. The second column indicates the relative abundance (# ESTs identified/2000 sequencing runs). The disease associated with mutations in each gene are shown in the the third column. The OMIM (Online Mendelian Inheritance in Man) entry number is shown in the fourth column.

Connexin 43 (GJA1), represented by 4 ESTs in ON, is the locus of oculodentodigital dysplasia (OMIM:164200), a disease whose clinical synopsis includes glaucoma. GJA1 is the major gap junction protein of astrocytes and there are data to suggest it may have a neuroprotective role in ischemia[26].

## Conclusion

The new EST analysis gives the first large scale overview of gene expression in the human ON. Previously available datasets described in Unigene as having ON origin seem to be misidentified or may include transcripts from other parts of the eye. For the NEIBank database, these Unigene or "dbEST" data have been combined (as NbLib0069 dbEST human "optic nerve" combined) and are available in a 'For The Record' section but are probably not a good representation of ON [see Additional File 1: Table S1]. In contrast, the new human ON cDNA library described here contains a convincing profile of axonal, oligodendrocyte and microglial markers and lacks significant contamination from other part of eye. Since ON is essentially a CNS white matter tract, this library is valuable for analysis of both genes generally expressed in white matter, compared with neuron soma, as well as for comparison between different CNS white matter regions. Many of the most abundantly expressed genes are associated with key ON functions, such as axonal growth, guidance, myelination and astrocyte function and are predicted to be expressed at high levels in a pure white matter CNS tissue.

A large number of mRNAs for genes known to be axonally transported in non-human CNS are also identifiable in the human ON library. These range from Vimentin (9/2000 or 0.45% of all sequenced ESTs) to individual ESTs such as Periredoxin 6 and Calreticulin. While a number of these mRNAs may also be expressed in intrinsic glial cells of the ON, it is also likely that many of them are axonally transported in human ON. Definitive proof of this activity is a relevant subject for a future study.

The relatively elevated levels of genes for inflammatory markers and clusterin may represent perimortem artifactual conditions of donors. Although many ion channel related genes are expressed in the ON, L-type calcium ion channels were observed at low levels. While detection of low abundance clones by EST is stochastic and absence of clones does not mean absence of expression, our RT-PCR and western results suggest that indeed the expression of this class of ion channel protein is lower in primates than in rodents. This finding may explain the difference in responsiveness to L-type calcium channel blockers in different species. This result suggests that further species comparisons of gene expression in ON may be valuable in development of ON therapeutic models.

Several genes that are the loci for inherited eye disease are expressed in ON and some of them, notably EFEMP1 and GJA1, are quite abundant. Previously, differential gene expression of a mitochondrially expressed gene (ND4) in a regional retinal pattern was correlated with Lebers hereditary optic neuropathy; where mutations in this gene correlate closely with a tissue region-specific dysfunction. Similarly, the current work suggests that differential gene expression may contribute to relative ON disease resistance or susceptibility, in both acquired and inherited diseases.

## Methods

### ON tissue

Human post-mortem tissue was obtained under University of Maryland Institutional Review Board (IRB) exemptions SB-019701 and SB-129901 Human ON tissue was dissected from freshly obtained 7 donor globes with attached ON. A detailed hospital medical history was obtained from each donor, but with masked identity, as per National Disease Research Interchange (NDRI) protocol (Table 6). No individuals with intrinsic ON disease, or any evidence of ocular infection were used. Monkey ON tissue was obtained from 8 euthanized rhesus macaques (4 male and 4 female; 4-6y/o). The ON sheath was removed from both human and rhesus ON. Rat ON tissue was obtained from 6 Sprague-Dawley young adult (60-90 d/o) male rats. Tissue was tissue quick frozen at -70°C until use. Additional tissues used for immunohistology were fixed in 4% paraformaldehyde-Phosphate buffered Saline (PF-PBS; pH 7.4) and either paraffin embedded or used for frozen section.

**Table 6:** Human donor tissue characteristics.

**Number**	**Age**	**Sex**	**Cause of Death**	**Time to dissection (hr)**
1	41	F	Liver abscess	39

2	62	M	Pneumonia	30

3	74	F	CHF	36

5	74	M	Prostate Ca.	48

4	76	F	Pulm. Emb.	30

6	80	M	Pneumonia	27

7	92	M	CVA	36

Optic nerve samples were obtained from 7 human donors (column 1). The age of each donor is shown in column 2, and their sex in column 3. Cause of death is indicated (column 4) and the time from demise to dissection, isolation and storage is shown in column 5.

### RNA isolation

Total human RNA was isolated using RNAzolB (Tel test Inc; Friendswood, TX). 100ug of total RNA was used for generating the human cDNA library. Poly (A+) RNA was obtained using an oligo [dT] cellulose column. Total rhesus and rat RNA were isolated using the Qiaprep kit (Qiagen; Darmstadt, Germany). A260:A280 ratios for total RNA were 1.8 or greater.

### Northern analysis

Northern analysis was performed to determine total RNA quality, prior to use. Ethidium bromide staining of 18s rRNA band was used to normalize total RNA loading following electrophoresis on denaturing formaldehyde-agarose (1.8%) gels [27]. Northerns for rhesus monkey (*Macaca mulatta*) eye tissues were prepared as described previously [28]. A cDNA for the human calcium channel protein was identified from the ON library. The insert was excised and labeled using a prime-it II kit (Stratagene systems, La Jolla, CA) and 32P-labelled dCTP. Northern blots were prehybridized in Hybrisol II (Oncor, Gaithersburg, MD) for 4 h, followed by hybridization with the specific radiolabelled cDNA probe at 63°C for 18 h. After hybridization, membranes were washed in 0.2× SSC, 0.1% SDS at 63°C and exposed to Kodax XAR or BMR photographic film for varying lengths of time at -70°C.

### cDNA Library Construction

A directionally cloned human ON cDNA library was constructed at Bioserve Biotechnology (Laurel, MD) using the Superscript II system (Invitrogen) and cloned into NotI/SalI sites of the pCMVSPORT6 vector (Invitrogen). Details of library construction can be found in [9]. The NEIBank code for the ON library is *nbj *and all clones are identified according to library, plate number and their position in 96 well plates, e.g.nbj01a01.

### cDNA Sequencing and Bioinformatics

Methods for sequencing and bioinformatics analysis are described in detail elsewhere [6]. Briefly, randomly picked clones were sequenced at the NIH Intramural Sequencing Center (NISC). Clones were sequenced from the 5' end. GRIST (GRouping and Identification of Sequence Tags) was used to analyze the data and assemble the results in web page format [10].

### Polymerase Chain Reaction (PCR)

PCR was used to validate alternative splice forms, obtain probes for hybridization, and to complete sequences. For sequence template, a sample of the complete cDNA library representing at least one million primary clones was amplified and plasmids isolated using reagents from Qiagen (Valencia, CA). PCR fragments were amplified using either Taq (Roche, Indianapolis, IN) or Elongase (Life Technologies, Gaithersburg, MD) polymerase systems and following the manufacturer's protocols.

Messenger RNA levels were quantified using real time quantitative (RQ) PCR, in a Biorad I-cycler. Single probes were analyzed using Syber green incorporation, and compared against an internal standard (cyclophilin B). Gene primers for cyclophilin B and two voltage gated dependent calcium channel subunits (CACNA1A; alpha 1A, P/Q type and CACNA1D; alpha 1D, L-type) were generated against conserved protein coding sequences present in all three (human, rhesus, rat) species. Primers used for CACNA1A were (human/forward: 5' ATG AAG CGT TCA GCC TCC GT, and rat/forward: 5' ATG AAG CGC TCA GCC TCC GT, and Human/rat reverse primer: 5' GA TTG GGT GGT CAT GCT CA. Primers used for CACNA1D were (Human:rat/forward: 5' TCC CTT CAG CAG ACC AAT ACC, and human:rat reverse: 5' TCC AGA CAC ATG CTC AAG GT. All primers generated equivalent size single product bands using the appropriate cDNA first strand templates.

### Immunohistochemistry, western analysis and confocal analysis

Fluorescent labeled donkey anti-rabbit, mouse and goat antibodies were purchased from Jackson immunoresearch (Pennsylvania). A rabbit antibody to QKI-5 was purchased from Bethyl Laboratories (Montgomery, TX). Mouse monoclonal antibody to GFAP (clone GA-5) was purchased from Calbiochem (La Jolla, CA). APC-1 antibody was purchased from Abcam.

Protein homogenates from freshly isolated male human, rhesus macaque, and Sprague-Dawley ON and brain were prepared using RIPA buffer as previously described [29]. Equal amounts of protein homogenate, measured by the Bradford reaction, was electrophoresed on 4-15% or 5% PAGE gels and transferred to nitrocellulose membranes. Membranes were blocked with I-block, and reacted with a mouse monoclonal primary antibody (N38/8) to a conserved region of the A1D subunit of the L-type calcium channel, purchased from Neuromab . Blots were stripped and reprobed with a rabbit polyclonal antibody to beta-actin (Sigma). Signals were detected using a commercially available fluorescent western analysis kit.

Human, monkey and rat ON tissues were fixed in Dulbecco's phosphate buffered saline (D-PBS)-4% Paraformaldehyde (PF). Fixed ON tissues were embedded in OCT, frozen on dry ice, and sectioned at 10 microns. Sections were reacted with primary antibodies, serially washed in D-PBS, reacted with the appropriate secondary labeled antibodies at 1:500 dilution, and examined using an Olympus 5 channel confocal laser microscope.

## Authors' contributions

SLB co-conceived of and participated in the design and coordination of the study, isolated and purified the ON tissues, performed much of the confocal microscopic analyses, specific molecular biological analyses and drafted the manuscript. GW co-conceived of and participated in the design and coordination of the study, generated the ON library and performed many of the sequence analyses and comparisons. KP generated the final genetic comparisons and multi-tissue comparisons, and YG performed the immunoassays, designed PCR primers, performed additional northern and real time PCR analyses and confocal microscopic analysis. All authors read and approved the final manuscript.

## Supplementary Material

Additional file 1 Supplementary table S1 Click here for file
