# Supplementary material for: Expressed sequence tag analysis of adult human optic nerve for NEIBank: Identification of cell type and tissue markers
Source: BMC Neurosci. 2009 Sep 24;10:121. doi: 10.1186/1471-2202-10-121 (PMC2762980; doi:10.1186/1471-2202-10-121)
Supplement: Additional file 1 — Supplementary table S1 [file 1471-2202-10-121-S1.DOC]

**Supplemental Table 1-Most abundant ESTs from Unigene human “ON” library**

ESTs known to be foreign tissue-specific are indicated in red.

| Description | Gene Id | # |
| --- | --- | --- |
| prostaglandin D2 synthase | 5730 | 15 |
| myelin basic protein (MBP) | 4155 | 14 |
| glutathione peroxidase 3 (plasma) (GPX) | 2878 | 13 |
| glial fibrillary acidic protein (GFAP) | 2670 | 9 |
| rhodopsin (RHO) | 6010 | 9 |
| clusterin (CLU) | 1191 | 8 |
| ribosomal protein S9 (RPS9) | 6203 | 7 |
| NADH dehydrogenase 1 beta 8 (NDUFB8) | 4714 | 7 |
| hexokinase 1 (HK1) | 3098 | 6 |
| elongation factor 1-gamma | 1937 | 6 |
| serpin peptidase inhibitor A3 (SERPINA3) | 12 | 6 |
| heat shock protein 1 (HSPB1) | 3315 | 6 |
| keratin 12 (KRT12) | 3859 | 6 |
| gelsolin (GSN) | 2934 | 5 |
| elongation factor 1 alpha 1 (EEF1A1) | 1915 | 5 |
| transferrin (TF) | 7018 | 5 |
| ferritin, heavy polypeptide 1 (FTH1) | 2495 | 5 |
| ribosomal protein L3 (RPL3) | 6122 | 5 |
| cytochrome P450 27A1 (CYP27A1) | 1593 | 5 |
| tubulin, alpha 1a (TUBA1A) | 7846 | 5 |
| glyceraldehyde-3-phosphate dehydrogenase (GAPDH) | 2597 | 4 |
| ribosomal protein L13a (RPL13A) | 23521 | 4 |
| stratifin (SFN) | 2810 | 4 |
| angiopoietin-like 7 (ANGPTL7) | 10218 | 4 |
| purine-rich element binding protein B (PURB) | 5814 | 4 |
| scaffold attachment factor B2 (SAFB2) | 9667 | 4 |
| ribosomal protein S2 (RPS2) | 6187 | 4 |
| ferritin, light polypeptide (FTL) | 2512 | 4 |
| serpin peptidase inhibitor F1 (SERPINF1) | 5176 | 4 |
| pyruvate kinase, muscle (PKM2) | 5315 | 4 |
| growth arrest-specific 6 (GAS6) | 2621 | 4 |
| ribosomal protein LP1 (RPLP1) | 6176 | 4 |
| opticin (OPTC) | 26254 | 4 |
| Wolf-Hirschhorn syndrome candidate 1 (WHSC1) | 7468 | 4 |
| alpha-transducin (GNAT1) | 2779 | 4 |
| solute carrier 68 (SLC6A8) | 6535 | 4 |
| creatine kinase, brain (CKB) | 1152 | 4 |
| elongation factor 1 delta (EEF1D) | 1936 | 4 |
| cystatin C (CST3) | 1471 | 4 |
| secretory carrier membrane protein 5 (SCAMP5) | 192683 | 4 |
| insulin-like growth factor binding protein 2 (IGFBP2) | 3485 | 4 |
| CD81 molecule (CD81) | 975 | 4 |
